# Clinical outcomes of arthroscopic rotator cuff repair: a retrospective comparison of double-layer, double-row and suture bridge methods

**DOI:** 10.1186/s12891-018-2244-y

**Published:** 2018-09-11

**Authors:** Hironori Kakoi, Toshihiko Izumi, Yasunari Fujii, Satoshi Nagano, Takao Setoguchi, Yasuhiro Ishidou, Setsuro Komiya

**Affiliations:** 1 Department of Orthopedic Surgery, Graduate School of Medical and Dental Sciences, Kagoshima University, 8-35-1 Sakuragaoka, Kagoshima City, 890-8520 Japan; 2 Health Service Center, National Institute of Fitness and Sports in Kanoya, 1 Shiromizu-cho, Kanoya, Kagoshima, 891-2393 Japan; 3 Near-Future Locomotor Organ Medicine Creation Course (Kusunoki Kai), Graduate School of Medical and Dental Sciences, Kagoshima University, 8-35-1 Sakuragaoka, Kagoshima City, 890-8520 Japan; 4 Department of Medical Joint Materials, Graduate School of Medical and Dental Sciences, Kagoshima University, 8-35-1 Sakuragaoka, Kagoshima City, 890-8520 Japan

**Keywords:** Arthroscope, Rotator cuff repair, Double-row method, Suture-bridge method, Delamination

## Abstract

**Background:**

The suture-bridge (SB) method has recently become the mainstream means of repairing full-thickness rotator cuff tears. However, in some patients the deep and superficial layers have moved in different directions because of delamination of their rotator cuffs. In such cases, a simple suture (double-layer, double-row [DD] method) is used to repair the superficial and deep layers separately. The purpose of this study was to analyze the clinical outcomes and re-tear rates of the DD and SB methods, with patients selected according to the condition of their torn cuffs.

**Methods:**

We retrospectively registered 74 patients with full-thickness rotator cuff tears that had been repaired arthroscopically, 35 shoulders by the DD and 39 by the SB method. Mean ages were 66.1 years in the DD and 62.9 years in the SB group. We evaluated clinical status before and after surgery (Japanese Orthopedic Association [JOA] scores) and re-tear rate. The Wilcoxon signed-ranks test was used to compare JOA scores and active ROM between before and after surgery in each group. Mann–Whitney’s U test was used for comparing JOA scores, active ROM, re-tear rates, size of tear, duration of follow-up, sex, and presence of subscapular muscle repair between the DD and SB groups. A hazard ratio of less than 5% was considered to denote significance.

**Results:**

JOA scores improved significantly in the DD and SB groups from preoperative means of 63.4 and 63.3 points, respectively, to postoperative means of 91.8 and 92.1 points, respectively. The active flexural ROM improved significantly from means of 110.1° and 100.0°, respectively, to postoperative means of 142.3° and 142.7°, respectively; the differences between groups were not significant. Re-tear occurred in 5.9% of the DD (two of 34 shoulders) and 7.9% of the SB group (three of 38 shoulders); its incidence did not differ significantly between the two groups.

**Conclusions:**

Both the DD and SB methods achieve satisfactory clinical outcomes that do not differ significantly. Our results suggest that careful selection of operative method on the basis of the delamination pattern in patients undergoing RCT may reduce the re-tear rate after utilizing the SB method.

## Background

Arthroscopic rotator cuff repair (ARCR) using suture anchors has evolved from a single-row (SR) to a double-row (DR) method, the latter being able to reproduce the foot print of the rotator cuff. However, after use of the DR method, mechanical stress is reportedly concentrated in the medial row, resulting in re-tear of the rotator cuff. In recent years, the suture bridge (SB) method has become the mainstream method for performing ARCR. This method spreads mechanical stress across the stump and increases tendon–bone interface pressure. Although several studies have reported excellent results of ARCR by the SB method, some authors have warned an associated risk of failure around the medial anchor, similar to that reported for the DR method. [1] Cho et al. reported failure of the medial rotator cuff when the medial row of mattress sutures passes through the rotator cuff in the SB method and discussed the possibility of strangulation and relatively quick necrosis of the rotator cuff tendon in the medial row. Therefore, although the mechanical characteristics achieved by the SB method are superior to those of other methods, it may not always be the best choice for full thickness rotator cuff (RCT) tears.

“Delamination” denotes horizontal separation of the tendon substance in a torn rotator cuff. Its incidence was recently shown to be as high as 82.8% of all RCTs. [2] The directions in which the deep and superficial layers retract may differ in delaminated cuffs. Sugaya et al. demonstrated that the double layer, double-row (DD) method of ARCR better achieves structural integrity of a delaminated cuff because it restores each layer to its original anatomical condition separately, [3] whereas with the SB method sutures are passed through the whole rotator cuff. Therefore, we consider that the SB method is contraindicated in patients with delaminated cuffs and different directions of retraction of the deep and superficial layers. However, no published studies have compared outcomes of the DD and SB methods after allocating patients on the basis of the characteristics of delamination.

The purpose of this study was to analyze the clinical outcomes and re-tear rates of the DD and SB method after basing patient selection on the condition of the torn cuff. Our hypothesis was that optimal selection of procedure on the basis of delamination characteristics would improve clinical outcomes and decrease re-tear rates after both methods of ARCR.

## Methods

### Patient selection

**Fig. 1:**
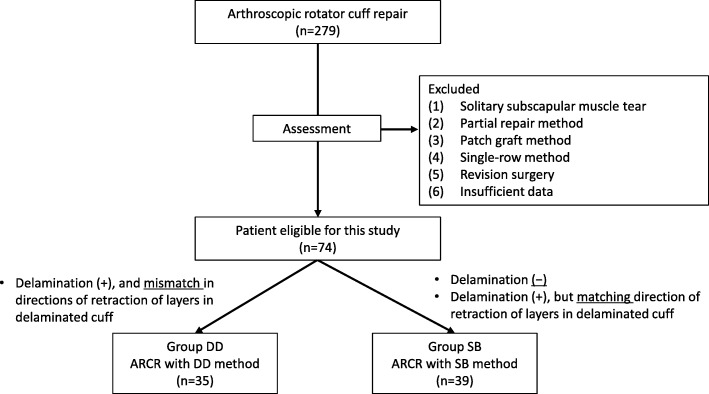
Flowchart showing study protocol

From January 2009 to June 2015, 279 ARCRs were performed in our department. SR, DR (including DD), partial repair, or patch graft methods were selected on the basis of the condition of the torn cuff. The SB method was performed from January 2011. The inclusion criteria for this retrospective study were: (1) full-thickness supraspinatus and/or infraspinatus tear that had been repaired by the DD or SB method of ARCR, and (2) follow-up for at least 12 months. The exclusion criteria included (1) solitary subscapular muscle tear, (2) partial repair method, (3) patch graft method, (4) SR method, and (5) revision surgery. After application of these criteria, 74 shoulders were enrolled in this study (Fig. 1).

Tear size was assessed at surgery according to the Cofield classification [4]. Delamination of the ruptured cuff and the direction of retraction of delaminated layers were also assessed by arthroscopic observation intraoperatively. Mean duration of follow-up was 17.1 months and 15.5 months in the DD and SB groups, respectively. No significant differences in age at surgery, sex, duration of postoperative follow-up, or tear size were identified between the two groups. Significantly more patients had undergone subscapularis repair in the SB than in the DD group. Patient characteristics are summarized in Table 1.

**Table 1:** Patient data

	Group DD (*n* = 35)	Group SB (*n* = 39)	*p* value
Age (y/o)	66.1 ± 6.0 (55–77)	62.9 ± 1.4 (44–78)	0.1718
Sex			
Male	26	28	0.8109
Female	9	11	
Follow-up period (months)	17.1 ± 6.6 (12–36)	15.5 ± 5.9 (12–37)	0.2553
Repair of subscapularis	22	33	0.0336
Tear size			
Small	2	3	0.4473
Medium	16	19	
Large	12	15	
Massive	5	2	
Delamination			
No	0	13	
Yes	35	26	
Retraction direction of the layers			
Matched	0	26	
Mismatched	35	0	

### Surgical technique

Surgery was performed under general anesthesia in the beach chair position.

Firstly, the size of the cuff tear was measured arthroscopically using a probe with a scale. The cuff stump was pulled out to determine whether the remaining cuff could sufficiently cover the greater tubercle. When it was not possible to cover the tubercle, the cuff was released and the foot print advanced 1 cm to the medial side of the humeral head. For tears considered repairable by the DD or SB method without excessive tension, one of these methods was chosen according to the presence of delamination and directions of retraction of the two layers as follows. If there was a mismatch in directions of retraction between the deep and superficial layers of a delaminated cuff, the DD method was utilized, that is, both deep and superficial layers of the rotator cuff were separately repaired using a standardized simple suture configuration. If the cuff had not delaminated or the direction of retraction of the deep layer could easily be aligned with that of the superficial layer of a delaminated cuff, the SB method was utilized. If it was considered impossible to repair the RCT without excessive tension by either the DD or SB method, either the SR, partial repair, or patch graft method was utilized.

### Postoperative management

An abduction pillow was used in all cases for 4–6 weeks after surgery depending on the tension of the repaired cuff. Passive range of motion (ROM) exercise was started three weeks postoperatively and active ROM exercises from 6 to 8 weeks postoperatively depending on the tension of the repaired cuff.

### Clinical assessment

**Fig. 2:**
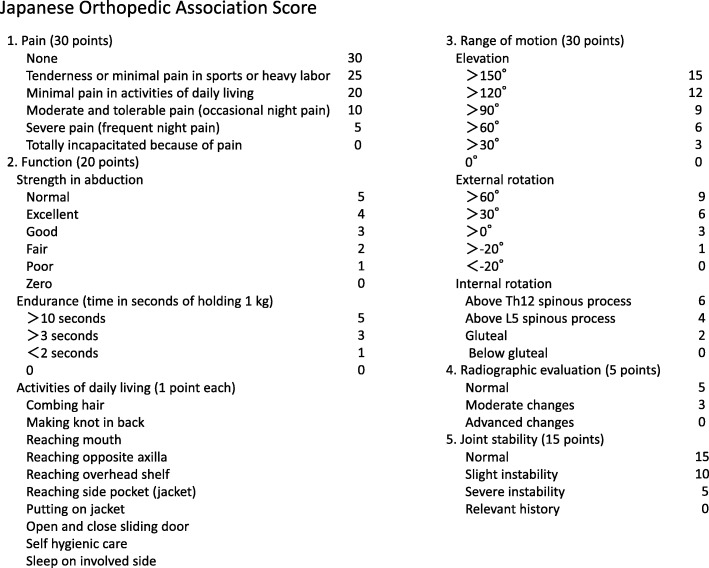
Japanese Orthopedic association scoring. This score comprises five items: pain, function, range of motion, imaging findings, and instability. A perfect score is 100 points

Clinical outcomes were evaluated preoperatively and at the last follow-up visit using Japanese Orthopedic Association (JOA) scores (Fig. 2) [5]. Active ROM (anterior elevation, external rotation, internal rotation) was also measured preoperatively and at the last follow-up visit. The extent of internal rotation was assessed at the height of the thoracic spinous process reached by a thumb. In accordance with the Ide et al. proposal, the first to fifth lumbar vertebrae were described as 13 to 17, the sacrum as 18, and the buttocks as 19 [6].

### MRI evaluation

The state of repair of the cuffs was assessed using MRI. MRI evaluation was available at the last follow up for 34 shoulders in the DD and 38 shoulders in the SB group. Re-tears were identified as MRI type 4 or 5 in accordance with the Sugaya classification [7].

### Statistical analysis

The Wilcoxon signed-ranks test was used for statistical analysis of JOA scores and active ROM between before and after surgery in each group. Mann–Whitney’s U test was used for comparing JOA scores, active ROM, re-tear rates, size of tear, duration of follow-up, sex, and presence of subscapular muscle repair between the DD and SB groups. A hazard ratio of less than 5% was considered to denote significance.

## Results

Total JOA score (maximum 100 points) improved significantly from a mean of 63.4 points preoperatively to a mean of 91.8 points at the final follow-up in the DD group. The SB group also showed significant improvement in JOA scores to 92.1 points from a mean of 63.3 points preoperatively. JOA scores did not differ significantly between the two groups, either before surgery or at final follow-up. Analysis of each factor in the JOA scores revealed significant improvements in pain (maximum 30 points), function (maximum 20 points), and ROM (maximum 30 points) in both groups. There were no significant differences between the two groups before surgery or at final follow-up for any JOA score factor (Table 2).

**Table 2:** Comparison of clinical results

	Preoperative		Final follow-up		*P*
	Mean ± SD (range)	*P* value	Mean ± SD (range)	*P* value	
JOA score total (Max. 100)					
Group DD	63.4 ± 8.4 (44–78)	0.9654	91.8 ± 7.2 (64–100)	0.9393	< 0.0001
Group SB	63.3 ± 9.2 (48–77)		92.1 ± 6.2 (73–100)		< 0.0001
Pain (Max. 30)					
Group DD	9.9 ± 3.5 (5–20)	0.9903	27.4 ± 3.3 (20–30)	0.4631	< 0.0001
Group SB	9.9 ± 3.7 (5–20)		26.7 ± 4.2 (15–30)		< 0.0001
Function (Max. 20)					
Group DD	14.2 ± 3.7 (6–19)	0.9957	19.1 ± 2.3 (11–20)	0.8389	< 0.0001
Group SB	14.0 ± 4.3 (5–20)		19.4 ± 1.1 (15–20)		< 0.0001
Range of motion (Max.30)					
Group DD	19.6 ± 5.8 (3–30)	0 .7858	25.0 ± 4.1 (17–30)	0.4733	0.0001
Group SB	20.1 ± 5.1 (6–30)		26.1 ± 2.7 (22–30)		< 0.0001

There were no significant differences in JOA scores between the two groups, either before surgery or at final follow-up

Active ROM (anterior elevation, external and internal rotation) was significantly improved in both groups, except for external rotation in the DD group. No significant differences in ROM were found between the two groups preoperatively or at final follow-up, except internal rotation at final follow-up. (Table 3).

**Table 3:** Comparison of active range of motion

	Preoperative		final follow-up		*P* value
	Mean ± SD(range)	*P* value	Mean ± SD(range)	*P* value	
Anterior elevation (°)					
Group DD	110.1 ± 40.7 (30–160)	0.1458	142.3 ± 15.17(100–180)	0.9520	0.0001
Group SB	100.0 ± 36.5 (20–150)		142.7 ± 15.1 (95–175)		<0.0001
External rotation (°)					
Group DD	38.3 ± 23.0 (−20 − 75)	0.2152	44.9 ± 21.5 (−5–80)	0.1395	0.0817
Group SB	45.4 ± 22.0 (0–80)		53.1 ± 16.3 (10–80)		0.0034
Internal rotation					
Group DD	14.2 ± 3.2 (7–20)	0.7521	12.3 ± 2.7 (7–19)	0.0340	0.0007
Group SB	14.4 ± 3.4 (7–19)		10.7 ± 3.6 (4–19)		<0.0001

There were no significant differences in ROM between the two groups, either before surgery or at final follow-up

Re-tear occurred in 5.9% of the DD group (two of 34 shoulders) and 7.9% of the SB group (three of 38 shoulders); its incidence did not differ significantly between the two groups (*p* = 0.739).

## Discussion

The initial fixation force achieved by the SB method is reportedly superior to that achieved by other repair methods using suture anchors because the SB method creates a larger contact area and higher pressure in the tendon–bone interface. In an experiment using fresh-frozen cadavers, Park et al. found that the contact area to footprint of a rotator cuff repaired by the SB method is approximately double that achieved by the DR method. They also reported that the contact pressure achieved by the SB method is 30% higher than that achieved by the DR method [8]. Additionally, Park et al. and Hatta et al. have reported that the SB method is superior to the DR method in terms of breaking strength, again according to experiments using fresh-frozen cadavers [9, 10]. Also in experiments using fresh-frozen cadavers, Steve et al. demonstrated that initial fixation strength is equivalent in that achieved by the SB and conventional trans-osseous methods. [11] Wenyong et al. found the SB method to be superior to the DR method regarding breaking strength of rotator cuffs in rabbits eight weeks after repair [12].

Consistent with these findings from basic research, many authors have reported good clinical outcomes for the SB method [13–16]. Several studies have compared the outcomes of the SB method with those of other methods of ARCR. Gary et al. analyzed ARCR for complete tears of a solitary supraspinatus tendon by the SR and SB methods and found the re-tear rate was significantly lower for the SB (7%) than the SR method (25%) [17]. Ide et al. analyzed clinical outcomes of ARCR using the SR and SB methods for anterosuperior rotator cuff tears and reported no significant difference in UCLA or JOA scores between the two methods. However, the re-tear rate was significantly lower for the SB (14%) than the SR method (32%). They also showed that functional tests of the subscapularis muscle such as the lift-off and belly press tests were performed better in the SB than the SR group [6].

Few studies have directly compared clinical outcomes between the SB and DR methods. Kim et al. compared clinical results between the DR and SB methods after ARCR for complete rotator cuff tears of anteroposterior diameter 1–4 cm in 42 shoulders. They showed that both methods achieved significantly improved average UCLA, ASES, and Constant scores; however, they found no significant differences between the two methods. In particular, the re-tear rate did not differ significantly between the DR (24%) and SB groups (20%). They concluded that the SB and DR methods are comparable with regard to patient satisfaction, clinical results, and re-tear rates [18]. Recent meta-analyses have reported that the re-tear rates of the DR and SB methods are equivalent [19, 20]. In agreement with those findings, we found no significant differences in clinical outcome, active ROM, or re-tear rate between the DD and SB methods, except internal rotation at final follow-up. It is not clear why internal rotation was better in the SB than the DD group. Kim et al. randomized patients to repair of delaminated rotator cuff tears by the DD or SB method and reported identifying no significant differences between the two groups in functional scores or ROM. However, pain scores were significantly lower in the DR than the SB group, whereas in our study the pain element of the JOA scores did not differ significantly between these groups. We believe that these apparent discrepancies are attributable to the selection criteria for the two methods. Kim et al. randomized their repair methods; thus, tears that were suitable for the DD method may have been repaired by the SB method [21].

There is an ongoing challenge to reduce re-tear rates after ARCR by employing different approaches. Healing of the repaired cuff affects re-tear rates. In this regard, Cho et al. have reported that cuffs repaired by SB have high re-tear rates (28.9%) and suggested the possibility of necrosis of the repaired tendon [1]. Recently published basic research on RCT in rabbits has shown impaired healing in the repaired cuff after the SB method, supporting this hypothesis [22]. In their histological study, more vessels and less fibrinoid deposition were observed in a parallel trans-osseous repair (PTR) group than in an SB group. Biomechanical strength did not differ at 1 week; however, it was significantly worse in the SB than the PTR group at 2 weeks. In the SB group, failure in the medial row occurred with significant frequency at 2 weeks. Therefore, although SB strongly compresses the tendon to the footprint of the rotator cuff, it may negatively affect re-vascularization or fibrosis of the cuff, resulting in impairment of healing. These findings also explain the mechanism of failure around the medial anchor after the SB method, which is similar to the failure after the DR method. We and others ascertain the directions of movement of layers of delaminated cuffs to determine whether to utilize the DD or SB method. Sugaya et al. precisely analyzed the pattern of delamination of RCTs and utilized the DD or SB method depending on the direction of retraction of the delaminated cuff [2]. They achieved a 7.6% re-tear rate in the two groups combined. In our study, in which we allocated delaminated cuffs on the basis of the direction of retraction (Fig. 1), we observed low re-tear rates for both the SB (7.9%) and DD methods (5.9%).

Our strategy for ARCR is to choose the optimal repair technique on the basis of the type of RCT. For instance, in the DR group the tears were mostly U- or L-shaped, which cannot be optimally repaired by the SB method. However, some authors have reported converting these types of tears to a crescent-shape as a first step and then repaired the resultant crescent tear by the SB method [23, 24]. As discussed above, the SB method is not always the best for ARCR because of both biomechanical strength and biological healing of the tendon. It is important to repair RCT by a method that does not produce excessive tension, this being achieved by careful assessment of the shape and delamination status of the torn rotator cuff.

Our study has some limitations. First, it was a small study, making it difficult to draw definite conclusions or prove our hypothesis. Second, the duration of follow-up was short. Third, the operator selected the operative method according to the condition of the torn cuffs. Finally, because we did not incorporate a control group without preoperative selection, we cannot draw the conclusion that patient selection according to the shape of the cuff tear can improve clinical outcome and decrease re-tear rate. Future studies with an appropriate control group may provide more stringent evidence concerning our hypothesis.

This study also had some strengths. First, all ARCR surgery was performed by a single surgeon. Second, to the best of our belief we are the first to report clinical results and re-tear rates for the SB and DD methods after allocating patients according to the preoperative condition of the ruptured cuff.

## Conclusions

Both DD and SB methods achieve satisfactory clinical outcomes that do not differ significantly. Our results suggest that careful selection of operative method on the basis of the delamination pattern in patients undergoing RCT may reduce the re-tear rate after utilizing the SB method.

## Abbreviations

ARCR: Arthroscopic rotator cuff repair
DD: Double-layer, double-row
DR: Double-row
JOA score: Japanese orthopedic association score
ROM: Range of motion
SB: Suture-bridge
SR: Single-row
